# Blueberry-Based Meals for Obese Patients with Metabolic Syndrome: A Multidisciplinary Metabolomic Pilot Study

**DOI:** 10.3390/metabo9070138

**Published:** 2019-07-10

**Authors:** Anatoly Petrovich Sobolev, Alessandra Ciampa, Cinzia Ingallina, Luisa Mannina, Donatella Capitani, Ilaria Ernesti, Elisa Maggi, Rita Businaro, Maria Del Ben, Petra Engel, Anna Maria Giusti, Lorenzo M. Donini, Alessandro Pinto

**Affiliations:** 1 Laboratorio di Risonanza Magnetica “Annalaura Segre”, Istituto per i Sistemi Biologici, CNR, via Salaria km 29.300, I-00015 Monterotondo, Italy; 2 Dipartimento di Chimica e Tecnologie del Farmaco, Sapienza Università di Roma, Piazzale Aldo Moro 5, I-00185 Roma, Italy; 3 Sezione di Fisiopatologia Medica, Scienza dell’Alimentazione ed Endocrinologia - Dipartimento di Medicina Sperimentale, Sapienza Università di Roma, Piazzale Aldo Moro 5, I-00185 Roma, Italy; 4 Dipartimento di Scienze e Biotecnologie medico-chirurgiche, Sapienza Università di Roma, Corso della Repubblica 79, 04100 Latina, Italy; 5 Dipartimento di Medicina Interna e Specialità Mediche, Policlinico Umberto 1 Sapienza Università di Roma, viale del Policlinico 151, I-00185, Roma, Italy; 6 Consiglio per la Ricerca in Agricoltura e l’Analisi dell’Economia Agraria (CREA), Ufficio Rapporti Istituzionali e Relazioni Internazionali, Via Po 14, 00198 Roma, Italy

**Keywords:** ^1^H-NMR, urine, cytokines, metabolic syndrome, blueberries

## Abstract

A pilot study was carried out on five obese/overweight patients suffering from metabolic syndrome, with the aim to evaluate postprandial effects of high fat/high glycemic load meals enriched by blueberries. Postprandial urine samples were analyzed by ^1^H-NMR spectroscopy after 2 and 4 h from ingestion to identify potential markers of blueberry intake. Significant decrease of methylamines, acetoacetate, acetone and succinate, known indicators of type 2 diabetes mellitus, were observed after the intake of meals enriched with blueberries. On the other hand, an accumulation of *p*-hydroxyphenyl-acetic acid and 3-(3’-hydroxyphenyl)-3-hydropropionic acid originating from gut microbial dehydrogenation of proanthocyanidins and procyanidins was detected. Real-time PCR-analysis of mRNAs obtained from mononuclear blood cells showed significant changes in cytokine gene expression levels after meals integrated with blueberries. In particular, the mRNAs expression of interleukin-6 (IL-6) and Transforming Growth Factor-β (TGF-β), pro and anti-inflammation cytokines, respectively, significantly decreased and increased after blueberry supplementation, indicating a positive impact of blueberry ingestion in the reduction of risk of inflammation. The combined analysis of the urine metabolome and clinical markers represents a promising approach in monitoring the metabolic impact of blueberries in persons with metabolic syndrome.

## 1. Introduction

In recent years, high attention has been paid to nutraceuticals and functional foods, capable of conferring health benefits to consumers in addition to their usual eating habits. In this context, a central role is being attributed to fruits of blueberry (*Vaccinium* L.) which, thanks to their composition in flavonoids and polyphenols, show anti-oxidant as well as anti-inflammatory activities, increasing the production of anti-inflammatory cytokines and decreasing oxidative stress and pro-inflammatory cytokines [1]. Numerous studies have investigated the action of blueberries in the modulation of inflammatory and antioxidant responses, attributable to the presence of high concentrations of anthocyanins, pro-anthocyanidins, phenolic acids and stilbenes [2]. Clinically it is well known that a diet rich in phytochemicals is associated with reduced cardiovascular risk, fewer events of stroke, myocardial infarction and cardiovascular death [3]. High consumption of specific whole fruits, particularly blueberries, grapes, and apples, has been significantly associated with a lower risk of type 2 diabetes [4]. In addition, epidemiological studies show a convincing link between antioxidant and anti-inflammatory properties of plant-derived polyphenolic compounds and their positive effect on metabolic syndrome [5], as well as their immunoregulatory role [6]. Dietary refinements may therefore provide a strategy to promote health in obese people as a means to treat oxidative stress (OS) and chronic inflammation [7]. 

Inflammation and OS are strongly interlinked, so that inflammation increases the production of reactive oxygen species (ROS) and vice versa, and either phenomena play a key role in the pathogenesis of the main chronic degenerative diseases, such as metabolic syndrome (MetS) and diabetes mellitus type 2 (DMT2). Overweight and obesity, particularly if associated with a sizable proportion of visceral adiposity, is characterized by a low-grade of chronic inflammation and an increased oxidative stress which contributes to the pathogenesis of MetS and other obesity comorbidities [8]. 

In this pilot study, conducted according to a crossover design, the effect of the consumption of high fat/high glycemic load (HF-HGL) meals including blueberries on the inflammatory state of overweight/obese patients with metabolic syndrome was investigated, using a ^1^H-NMR-based metabolomics approach [9,10,11] together with assays of inflammatory stress (real-time PCR). It is well known that in the hours following consumption of a meal there is an increase in serum levels of several inflammatory mediators [12]. The effects of blueberry addition to a HF-HGL meal were monitored at two and four hours after the meals. These time points were chosen taking into account the results reported in previous studies. Anti-inflammatory activity of strawberries has proven to be delayed versus the antioxidant activity of blueberry. The anti-oxidant activity of blueberries appears within three hours after consuming a high carbohydrate, low fat breakfast [13], whereas the effects of strawberries on plasma inflammatory markers after consuming a high carbohydrate, moderate fat meal were not apparent until six hours [14].

## 2. Results and Discussion

For this pilot study, five patients with MetS were recruited and provided with a single meal with and without blueberry addition. The essential anthropometric characteristics (age, gender, body mass index, waist circumference, body composition) of the patients are reported in Table 1. 

### 2.1. Anthropometric Characteristics

All subjects met at least three criteria for the diagnosis of MetS according to the NCEP–ATP III (2001) (waist circumference for men > 102 cm and for women > 88 cm, triglycerides ≥ 150 mg/dL, High-density lipoprotein (HDL)-cholesterol for men < 40mg/dL and for women < 50 mg/dL, blood pressure ≥ 130/80 mm/Hg, fasting glucose ≥ 110 mg/dL) [15]. According to the exclusion criteria of the study, none of the recruited subjects were diagnosed with diabetes mellitus or taking antidiabetic drugs. All subjects had a waist circumference higher than the cut-offs provided by ATP-III, four subjects had low HDL-cholesterol level, two subjects suffered from hypertension, three subjects had hypertriglyceridemia, and two subjects suffered from impaired fasting glycemia (IFG).

**Figure 1:**
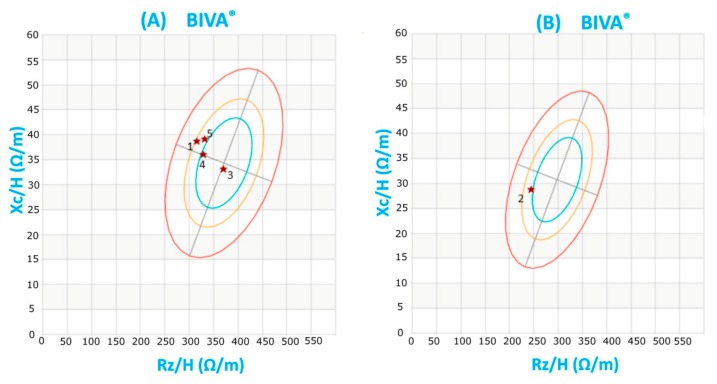
Subjects’ Bioelectrical Impedance Vector Analysis (BIVA): The graphical representation (R-Xcgraph) known as BiaVector® represents resistance (Rz) and reactance (Xc) normalized by height. Rz and Xc were measured at 50 kHz. The three concentric ellipses, proceeding from the center towards the periphery of the graph, represent the 50th, 75th and 95th percentiles of the BIVA distribution in a reference population. Each subject was plotted in the R-Xcgraph. BIVA shows that all subjects fall within the 75th percentile ellipse for the reference population, (**A**) females and (**B**) males, demonstrating the absence of significant hydration and body composition abnormalities.

Two subjects were obese (BMI ≥ 30 kg/m^2^) and three were overweight (25 kg/m^2^ ≤ BMI ≥ 30 kg/m^2^); waist circumference was more than 88 cm in all female subjects, attesting intra-abdominal fat accumulation. Applying the vector analysis method (bioelectrical impedance vector analysis, BIVA®) to the bioelectrical impedance measures (resistance R and reactance Xc), all testing persons fell inside the 75% tolerance ellipse for the reference population, showing a normal body hydration (total body water, extra-cellular water, intra-cellular water) and composition (fat mass, fat free mass and body cell mass) for BMI (Figure 1) [16].

No subject met the indexes or the criteria of body composition for the diagnosis of sarcopenic obesity: the fat free mass index (FFMI) was > 75th percentile in the Italian population (M = 21 kg/m^2^ and F = 17.2 kg/m^2^) [17]; the FM (kg)/FFM (kg) ratio was lower than the 75th percentile adjusted for sex, age and BMI for the non–Hispanic white individuals identified by Xiao et al. (2018) [18]. 

Moreover, the skeletal muscle mass index (SMI), the appendicular skeletal muscle mass/height^2^ (ASMM/height^2^) and the appendicular skeletal muscle mass/weight were calculated to compare them with the specific reference values for sex and age available in the literature, even with the limits of the measurements performed with Bioelectrical Impedance Analysis (BIA) [19,20]. 

### 2.2. Blueberry Meals

A high fat/high glycemic load meal in the presence or the absence of blueberry addition was used. According to the literature, intake of high energy density meals, particularly rich in saturated fats, induce an acute postprandial inflammatory response, approximately 60 min after consumption [21]. 

The administered meal (Table 2) was characterized by a high energy density, due to the high lipid component (>30% of meal total energy) mainly consisting of saturated fatty acids; also the glycemic load of the meal was rather high according to the University of Sydney classification system (low glycemic load: ≤ 10; medium glycemic load: 11 to 19; high glycemic load: ≥ 20) [22]. Both high-fat meals and high glycemic loads, comparable to the features of the Western dietary patterns, induce a postprandial inflammatory response, which is more evident in obese subjects and persons suffering from type 2 diabetic [23]. The effects of this meal could be partly countered by the intake within the meal of blueberries.

### 2.3. NMR Data and Statistical Analysis

**Figure 2:**
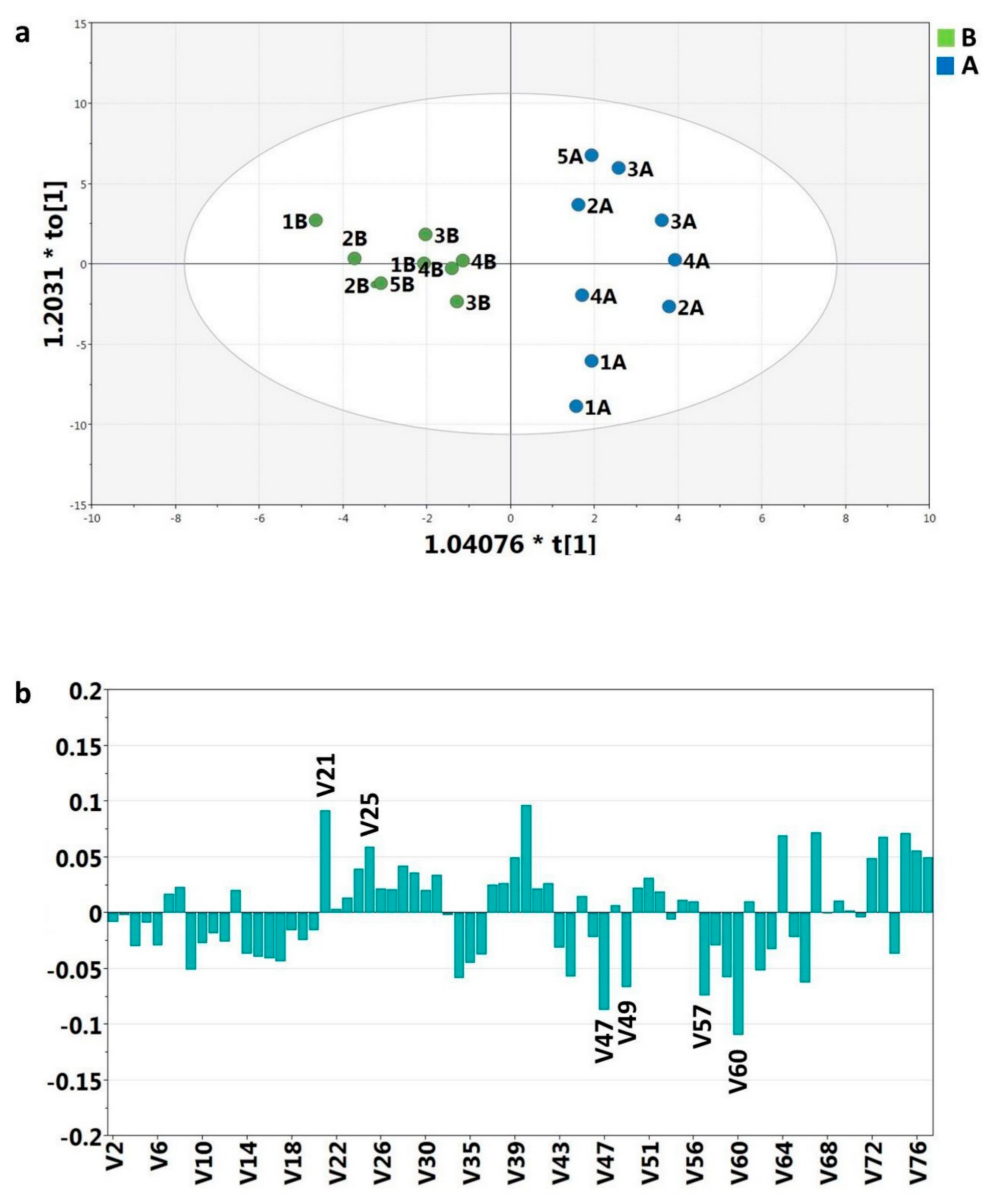
(**a**) Orthogonal partial least-squares-discriminant analysis (OPLS-DA) scores plot. Postprandial urines with (A) and without (B) blueberries of five patients two and four hours after intake. R^2^X_cum_ = 0.304 (fraction of variance cumulative in the X matrix), R^2^Y_cum_ =1.0 (fraction of cumulative variance in the Y matrix); (**b**) coefficients plot of the 75 variables from ^1^H-NMR spectra.

^1^H-NMR spectroscopy was used to compare the ^1^H spectra of patients’ urines after meals with and without blueberry addition. The ^1^H-NMR spectra were analyzed according to the methods described in recent literature [24,25]. In particular, spectra were divided in 75 bins which were integrated and normalized (see Material and Methods section). The 75 normalized integrals were considered as variables for statistical analyses. Orthogonal partial least-squares-discriminant analysis (OPLS-DA) was applied to explore possible effects of blueberry intake. The score plot for the two-component model (one orthogonal and one predictive) reveals a good separation between postprandial urines with and without blueberry intake according to explained variance (R^2^ = 0.852) and predictive capability of the model (Q^2^ = 0.41) (Figure 2a). 

According to variable coefficients (Figure 2b) the most significant discriminatory bins for the postprandial urine differences were V60, V21, V40, V47, V57, V49, V67, V73, V25.

The variable importance derived from the OPLS-DA model was used to screen for important metabolites, whose significance was further verified through analysis of variance (ANOVA).

The ANOVA data were generally consistent with those derived from the OPLS-DA model, with the exception of V40, V67, V73, which demonstrated no significant difference between the two groups in exam.

In particular, the ANOVA results showed that only six variables (Table 3) turned out to be significant to differentiate urinary samples after meals with and without blueberries. These six variables were assigned, according to current literature [26,27] to acetoacetate/acetone (V60), succinate (V57), dimethylamine (V49), trimethylamine (V47), 3-(3’-hydroxyphenyl)-3-hydropropionic acid/*p*-hydroxyphenyl-acetic acid (V25), and 3-(3’-hydroxyphenyl)-3-hydropropionic acid/*p*-hydroxyphenyl-acetic acid/histidine (V21) (Table 3). 

**Figure 3:**
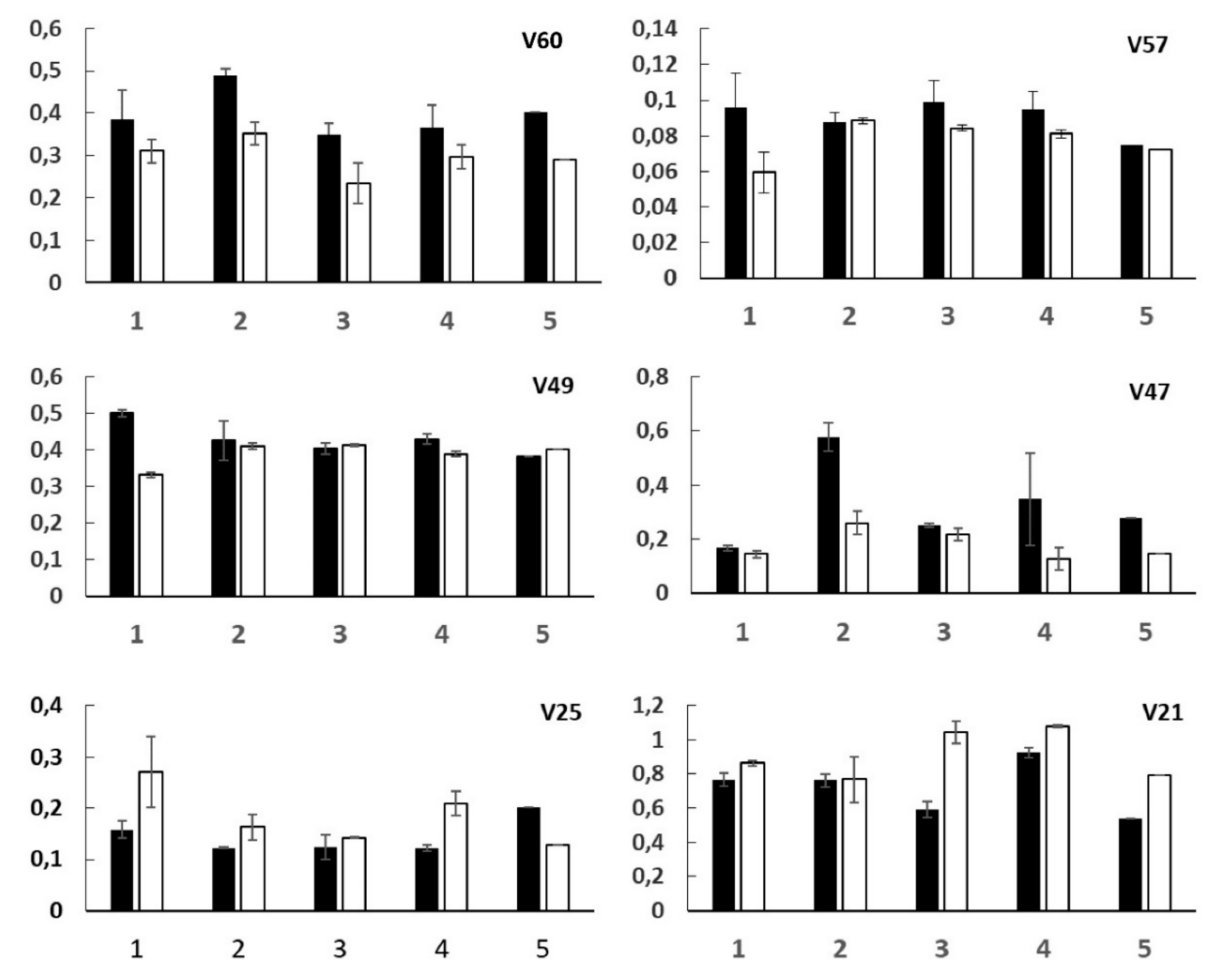
Histograms relative to the mean values (after 2 and 4 h) of bin variables selected by ANOVA analysis for five patients (1, 2, 3, 4, 5). Black and white bars are due to meals without and with blueberries, respectively. Standard errors are also reported.

In order to monitor the trend of the bin variables related to specific compounds, after meals with and without blueberries, variables histograms were reported in Figure 3. Each variable is discussed separately.

#### 2.3.1. V60—Ketone Bodies 

An extremely significant decrease of acetoacetate and acetone levels was observed in urinary samples after meals with blueberries compared to meals without blueberries (Figure 3). The antioxidants contained in blueberries probably slow down the ketone production and oxidation/pro-inflammatory processes [28]. Elevated ketone body levels may be a physiological consequence of enhanced ketogenesis, eventually due to a ketogenic/fat-rich nutrition or a glucose defective absorption or an insulin deficiency [29].

In diabetic patients, ketone body concentration is high [30] and their detection in urine is a danger marker which suggests a poorly controlled diabetes [31]. In particular, the increased levels of acetoacetate, acetone, acetate as well as n-butyrate, α-hydroxy-*n*-butyrate, and β-hydroxybutyrate, derive from β-oxidation process [32]. The insulin intake gives rise to a lowering of the ketone bodies values [28]; the intake of meals with blueberries goes in the same direction. 

#### 2.3.2. V57—Succinate and Tricarboxylic Acid (TCA) Cycle 

Lower levels of succinate in urinary samples after the meals with blueberries (Variable 57, Figure 3) with respect to meals without blueberries were observed. Succinate is an intermediate of tricarboxylic acid cycle in the glycolysis pathways. It is important to note that pre-diabetic and diabetic diseases can also lead to alterations in the TCA cycle intermediates reflecting either systemic stress produced by hyperglycemia or local effects on kidney tubular transport [32]. Increased excretion of TCA cycle intermediates, such as succinate, have been also observed in the urine of diabetic rats [33]. 

Moreover, outside the TCA cycle, new biological roles for succinate have been demonstrated as a key signal in multiple cellular functions, including the transcription factor hypoxia-inducible factor-1α (HIF-1α) stabilization, the succinate and succinate receptor 1 (SUCNR1, termed G protein coupled receptor 91) signaling during inflammation and the post-translational modification (PTM) of proteins by succinylation [34].

The low levels of succinate found in urine samples after meals with blueberries can, therefore, suggest the effect of blueberries in the regulation of glucose metabolism and inflammatory pathways.

#### 2.3.3. V49 and V47—Dimethylamine (DMA) and Trimethylamine (TMA)

DMA (V49) and in particular TMA (V47) levels significantly decreased (Figure 3) after meals with blueberries. As previously reported [35], methylamines, important osmoregulatory compounds, are produced from choline, introduced with the diet, that give rise to DMA and TMA by specific gut microflora [33,36]. Moreover, a change in the balance of methylamine metabolism with an increment in TMA concentrations was observed in diabetic rats [33]. 

Therefore, our results suggest the effect of the blueberries on gut microflora population and, consequently, on the regulation of methylamine metabolism.

#### 2.3.4. V25 and V21—*p*-Hydroxyphenyl-Acetic (HPA) and 3-(3’-Hydroxyphenyl)-3-Hydropropionic (HPHPA) Acids

The levels of *p*-hydroxyphenyl-acetic and 3-(3’-hydroxyphenyl)-3-hydropropionic acids increased in patients’ urine after blueberry meals intake (Figure 3), in particular after four hours. Interestingly, in the case of patient 5, whose urine was taken after only two hours, no increase of V25 was observed. 

*p*-Hydroxyphenyl-acetic and 3-(3’-hydroxyphenyl)-3-hydropropionic acids are produced by gut microbial dehydrogenation of some bioactive compounds such as proanthocyanidins and procyanidins, present in elevated quantity in blueberry fruits [2]. These microbial metabolites may contribute to the health promoting properties of proanthocyanidins in vivo [37].

Previous research suggested that procyanidins prevented or alleviated type 2 diabetes in part by inhibiting enzymatic starch digestion [38]. 

### 2.4. Pro- and Anti-Inflammatory Cytokines: Real-Time Quantitative PCR Analysis

It is well known that in the hours following the consumption of a meal there is an increase in serum levels of several inflammatory mediators [12]. For this reason, we set out to measure the pro- and anti-inflammatory cytokines modulation following high fat/high glycemic load meals in the presence or in the absence of blueberry.

mRNAs for pro-inflammatory cytokines, including Interleukin (IL)-1β, IL-6, Tumor Necrosis Factor (TNF) -α, and for anti-inflammatory cytokines, including IL-4, IL-10 and Transforming Growth Factor (TGF)-β obtained from blood mononuclear cells, were analyzed by real-time PCR. 

**Figure 4:**
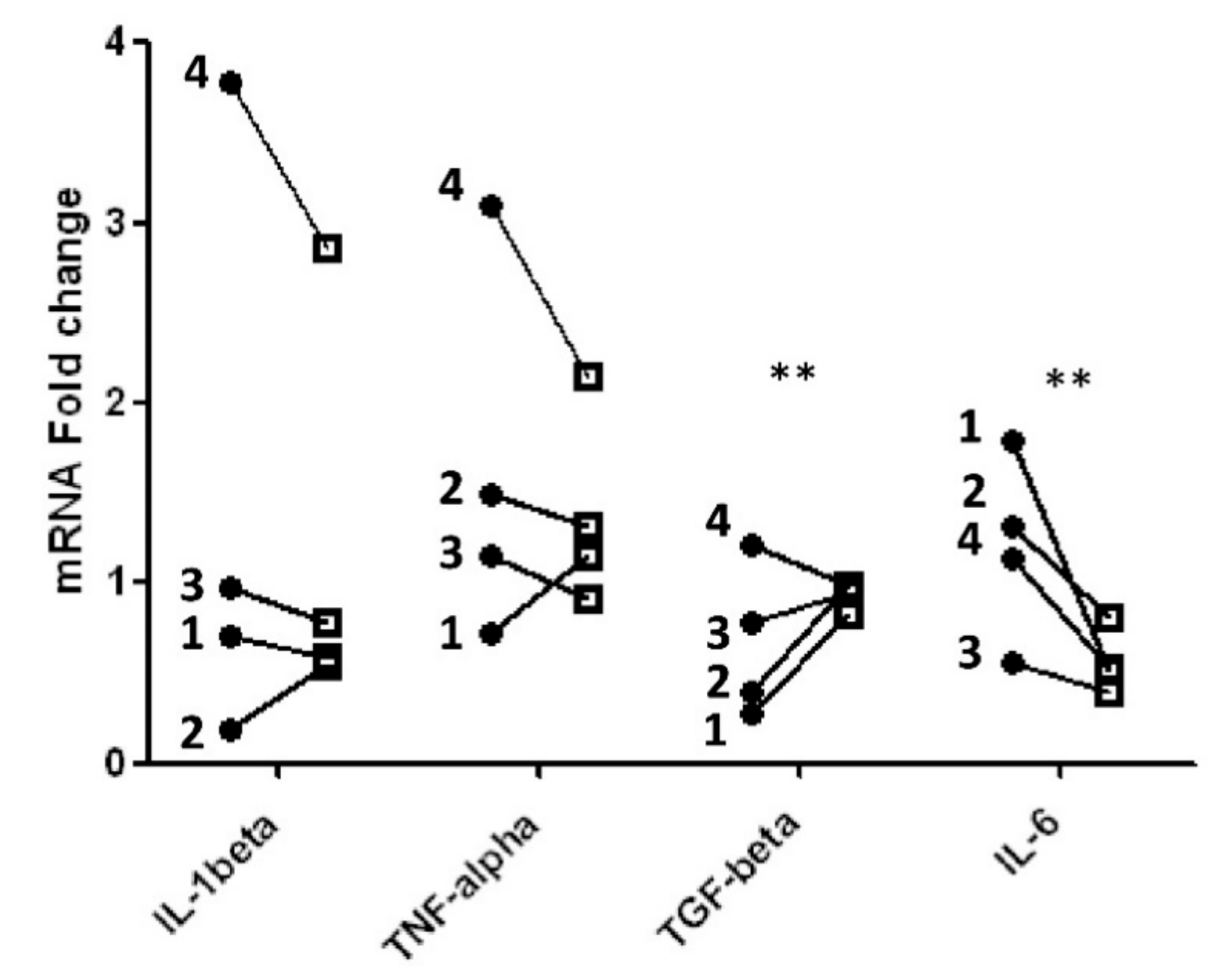
Fold change in cytokine gene expression level after feeding meals without (black circle) and with (black square) blueberries relative to gene expression levels detected before meals in different subjects (1, 2, 3, 4); ** Significant differences (*p*-value < 0.005).

As shown in Figure 4, mRNA levels purified from blood mononuclear cells obtained after meal consumption were compared to mRNA levels isolated from the same cells obtained before meal consumption (time 0). Values are reported as fold change detected in mRNA levels obtained after meals compared to time 0 (before meal). We compared variations in cytokine levels following meals containing blueberries with the respect to meals without blueberries, in order to ascertain the putative anti-inflammatory effect of blueberries added to high fat meal. 

Significant differences (*p* < 0.005), as reported in Table 4, were detected for IL-6 and TGF-β mRNAs: IL-6 mRNA significantly decreased, whereas TGF-β mRNA significantly increased after blueberry ingestion. The variations observed in IL-1β mRNAs obtained after consumption of meals with or without blueberries were different in the single patients. In three patients, blueberry intake gave rise to a decrease in IL-1beta however, the effect was different from patient to patient. Conversely, in patient 2 a weak increase of IL-1β mRNA two hours after a meal supplemented with blueberries was observed. A similar result occurred for TNF -α, as depicted in Figure 4. Our data are reinforced by previous results showing that blueberry extract added to lipopolysaccharide (LPS)-stimulated microglia induce a significant decrease of IL-1β and TNF -α released in the culture supernatants [39].

IL-6 significantly decreased four hours after meals in agreement with previous studies [20].

Moreover, polyphenols from blueberries modulated the mRNAs synthesis of IL-1beta and IL-6 in RAW264.7 macrophages stimulated by LPS, but with different kinetics [40]. 

Kinetic studies on the transcription of mRNA and secretion of IL-6, IL-1, and TNF -α revealed that TNF -α mRNA transcription and cytokine production occur very rapidly. TNF -α mRNA accumulation peaks 1–2 h after stimulation of murine macrophages with LPS and maximum concentrations of cytokine are found in supernatants collected after 2–4 h of culture. 

IL-6 bioactivity peaks between 8 and 12 h, whereas maximum concentrations of IL-1 bioactivity were not detected in supernatants from stimulated cells collected prior to 12 h of culture [41]. Therefore, it is possible that a significant decrease for IL-1beta would be observed only in blood samples obtained at a later stage.

Considering the mRNA levels for the anti-inflammatory cytokines, TGF-β significantly increased after a meal containing blueberries in comparison to a meal without blueberries. IL-4 and IL-10 were undetectable before the meals and became barely detectable after the meal consumption, at the limit of test sensitivity (data not shown). This behavior may be due to their different kinetics of synthesis, since they peak 10 hours after stimulation [42]. 

Our data reveal the temporal kinetics of pro- and anti-inflammatory signaling events that may be important therapeutic targets for inflammatory diseases.

The anti-inflammatory activity of blueberries has been demonstrated in several in vitro cellular models [39,43,44] and experimental animal models [45,46,47,48,49,50], while few studies performed on humans are available [1,51,52]. 

Blueberry phenolic acids mixture was found to show anti-inflammatory activities by inhibiting the nuclear factor-κB (NF-κB) activation and the production of inflammatory cytokines (TNF-α and IL-6) induced by LPS in RAW-Blue cells [53]. 

A study analyzing the anti-obesity effect of blueberries in a murine model proved that the consumption of a diet enriched with blueberries significantly led to decreased body weight gain in the presence of a high fat diet, reducing at the same time serum and hepatic lipid levels. Moreover, the expression of several pro-inflammatory cytokines was decreased [45]. Another study dealing with age-related alterations showed that mice fed a high fat diet develop memory deficits which can be recovered by adding blueberries to the diet and that fewer microglia were detected in the brains of mice fed a high fat diet containing blueberries compared to mice fed low fat and high fat diets without blueberry addition. Analysis of mRNAs obtained from mice hippocampus showed that brain-derived neurotrophic factor (BDNF) levels were higher and the number of neural precursors cells was greater in the hippocampus of mice fed high fat diet plus blueberry compared to mice fed high fat diet [43].

Albeit, several publications suggested that the anthocyanins are promising dietary bioactive compounds in prevention and treatment of DMT2 [54,55,56] and the management of MetS [57,58,59], the results of the studies on the blueberry anti-inflammatory effects in these chronic diseases are contradictory. This is despite the evidence that metabolically triggered chronic low-grade inflammation (metaflammation) is one of the main mechanisms responsible for insulin resistance [23]. Recently, literature reviews on the blueberry anti-inflammatory effects were published [60,61,62]. The authors conclude that the discordant findings may be explained by the heterogeneity in the study design and require further investigation (randomized double-blind clinical trials), more robust biomarkers of inflammation (or pattern of pro and ant-inflammatory biomarkers), with appropriate sample size, duration of exposure, exploration of dose–response relationships. A recent six-week randomized, double-blind, placebo-controlled and parallel arm clinical intervention trial demonstrated that blueberries attenuate oxidative stress and inflammation in adults with MetS, decreasing monocyte gene expression of TNFα, IL-6, TLR4 and reducing serum granulocyte macrophage colony-stimulating factor (GMCSF) in MetS subjects when compared to the placebo treatment (*p* ≤ 0.05) [63]. 

Furthermore, the mechanisms underlying blueberry effects, such as reduction of oxidative stress and inflammation, improvement of endothelial dysfunction, regulation of cholesterol accumulation and trafficking, along with potentially influence of gut microbiota, were discussed in a recent review showing the health benefits related to blueberries consumption [64]. 

## 3. Materials and Methods

### 3.1. Experimental Design

Five subjects (reported as Subject 1, Subject 2, Subject 3, Subject 4 and Subject 5) were enrolled for the study at the ambulatory of Internal Medicine and Metabolic Diseases of the Department of Internal Medicine and Medical Specialties of “Sapienza” University of Rome, Italy. The subjects were aged between 26 and 61 years and their body mass index (BMI) ranged from 28 to 40 kg/m^2^ (Table 1); all subjects were diagnosed with MetS, according to the NCEP–ATP III criteria [15]. The exclusion criteria from the study were: diabetes, cancers and/or chronic inflammatory diseases, pregnancy, lactation, smoking, alcoholism, vitamin or antioxidant supplement intake during the month preceding the study, use of drugs such as statins, anti-diabetics or the chronic use of nonsteroidal anti-inflammatory drugs (NSAIDs); allergy, food intolerance or adverse reaction following the assumption of blueberries. All test persons were asked to sign informed consent to participate in the study. According to the crossover study design, each patient consumed two meals with similar macronutrient composition, meal “A” with blueberries fresh fruit (150 g) added and meal “B” without blueberries, interspersed at a distance of 7–10 days (Table 2). The amount of blueberries (150 g) administered with meal “A” has been established on the basis of previous studies [14,65]. The composition of the two meals, “A” and “B”, is reported in Table 2. The composition of the meals was analyzed using the software Dietosystem Terapia Alimentare© release 15.00.06 (DS Medica Srl Milan, Italy).

Urine and plasma samples of each patient were taken at the Department of Experimental Medicine, Section of Medical Physiopathology, Nutrition Science and Endocrinology, Sapienza University of Rome, Italy. The following time points for sampling were utilized: in the morning before breakfast after overnight fasting (Time sampling = 0), after two (Time sampling = 1) and four hours (Time sampling = 2) from lunch. The samples were collected into pre-labeled sterile cups, and were immediately frozen and stored at –80 °C until the analysis.

### 3.2. Body Composition Analysis (BCA)

All the recruited subjects underwent the following measurements by a skilled operator, conforming to the procedures described in the Anthropometric Standardization Reference Manual [66,67]: body weight was measured to the nearest 0.1 kg through a standard column body scale (SECA, Hamburg, Germany); body height (using a rigid stadiometer—SECA, Hamburg, Germany) and waist circumferences (using a measuring tape) were determined to the nearest 0.1 cm.

### 3.3. Bioelectrical Impedance Analysis (BIA)

Whole-body impedance components, impedance (*Z*), phase angle (*PhA*), resistance (*R*) and reactance (Χc) were measured with the single-frequency 50 kHz analyzer NUTRILAB (AKERN Bioresearch SRL, Pontassieve, Florence, Italy). The measurements were undertaken following standardized procedures [68]. The external calibration of the instrument was checked with a calibration circuit of known impedance value. Fat free mass (FFM), fat mass (FM), total body water (TBW), extra-cellular water (ECW), intra-cellular water (ICW), and body cell mass (BCM) were obtained from bioelectrical data, using the gender-specific prediction equations provided by software Bodygram Plus 1.2.2.8. The fat free mass index (FFMI) and the fat mass index (FMI) were calculated through the normalization for height of FFM (kg) and FM (kg) obtained by the BIA: FFMI = FFM /height^2^ (kg/m^2^) and FMI = FM /height^2^ (kg/m^2^). Skeletal muscle mass (SM) was estimated by the Janssen equation [69] and the skeletal muscle mass index was calculated by dividing SM by height (m^2^); appendicular skeletal muscle mass (ASMM) was estimated by the Kim equation [70]. The measures are summarized in Table 1.

Whole-body bioimpedance vectors were analyzed by the RXc graph method [16] using the software Bodygram Plus 1.2.2.8. Each subject was plotted in the tolerance ellipses (50%, 75% and 95%) of the reference population (BIVA®). The BIVA point graph indicated that subjects fell mostly inside the 75% tolerance ellipse for the reference population (Figure 1), showing hydration and body composition within the normal range for the degree of body weight excess.

### 3.4. NMR Data Acquisition

For NMR sample preparation, an optimized standard protocol for NMR analysis was used [24,25]. First, frozen samples were thawed at room temperature (20 to 30 min). Completely thawed urine samples (taken two and four hours after lunch) were shaken before use, 630 µL was drawn, and centrifuged at 6000 *g* for 5 min at room temperature. Supernatant was added to 70 µL of potassium phosphate buffer (1.5M KH_2_PO_4_ phosphate buffer in D_2_O, pH 7.4) containing 0.1% sodium trimethylsilyl [2,2,3,3-^2^H_4_] propionate (TSP) and 0.2% of sodium azide. The entire aliquot of 700 µL of the mixture was put into a 5-mm NMR tube.

^1^H NMR experiments were recorded at 27 °C on a Bruker AVANCE 600 spectrometer operating at the proton Larmor frequency of 600.13 MHz (B_0_ = 14.1 T). For each urine sample, a 1D NMR spectrum was acquired with water peak suppression during relaxation delay using the noesygppr1d pre-saturation pulse sequence from the Bruker pulse library. The ^1^H spectra of the urine samples were acquired using the following conditions: number of scans 256, π/2 pulse ~ 15 μs, time domain 32 K data points, relaxation delay plus acquisition time 6.10 s, and spectral width 14 ppm. Total acquisition time for each sample was 27 min. ^1^H NMR spectra were obtained by the Fourier transformation of the free induction decay, applying an exponential multiplication with a line-broadening factor of 1 Hz and a zero filling (size = 64 K) procedure. ^1^H NMR spectra were manually phased. Chemical shifts were referenced internally to the singlet methyl resonance of TSP at 0.00 ppm. The baseline was manually corrected using the polynomial baseline correction routine in the Bruker TopSpin software (version 1.3). Signals were assigned using literature data, including freely available databases like Humane Metabolome Database [71]. 

Each ^1^H-NMR spectrum in the range between 0.2 and 10.00 ppm was segmented into 75 chemical shift bins (Supplementary, Table S1), and the corresponding spectral areas were integrated manually. Regions between 4.90–4.65 ppm and 6.00–5.54 ppm containing residual water and urea signals were removed [24]. The integrals were normalized according to probabilistic quotient normalization approach [72] and used as variables in ANOVA and OPLS-DA analyses. Normalization was carried out to account for different dilutions of urine samples, by scaling the spectra to the same virtual overall concentration. Statistica software package for Windows (1997; edition by Statsoft) and SIMCA (Version 15) software respectively were used for ANOVA and OPLS-DA analyses.

### 3.5. Real-Time Quantitative PCR Analysis

Blood samples added with sodium citrate were centrifuged at 1200 rpm 10 min at 4 °C. The top layer containing plasma was recovered and stored at −80 °C for subsequent measurements of plasma cytokines by ELISA. The buffy coat layer was incubated with 1mL lysis buffer (Buffer EL-Erythrocyte lysis buffer cat. n. 79217, Quiagen) for 20 min at 4 °C in order to eliminate residual erythrocytes. Samples were then centrifuged at 1200 rpm for 10 min at 4 °C, and washed twice with PBS. The pellet was then suspended in 700 μL Qiazol (Qiagen cat. 1023537) and processed for real-time quantitative PCR analysis.

Total RNA was extracted from the mononuclear cells using the miRNeasy Micro kit(50) (Qiagen, Hilden, Germany) and quantified using NanoDrop One/OneC (Thermo Fisher Scientific, Waltham, MA, USA). cDNA was generated using the High-Capacity cDNA Reverse Transcription kit (Applied Biosystem, Foster City, CA, USA). Quantitative real-time PCR (qPCR) was performed for each sample in triplicate on an Applied Biosystems 7900HT Fast Real-Time PCR System (Applied Biosystem, Cheshire, UK) through the program SDS2.1.1 (Applied Biosystem, Foster City, CA, USA) using the Power SYBR® Green PCR Master Mix (Applied Biosystem, Foster City, CA, USA). The primers for PCR amplification reported in the Table 5 were designed with UCSC Genome Browser. A comparative threshold cycle (CT) method was used to analyze the real-time PCR data, where the amount of target, normalized to the endogenous reference of hGAPDH (ΔCT) and relative to the calibrator of untreated control (ΔΔCT), was calculated by the equation 2 -ΔΔCT as previously described in literature [73] (Table 5).

GraphPad Prism software package (Version 6.0) was used for two-way ANOVA analysis, followed by uncorrected Fisher’s LSD.

### 3.6. Ethical Statement

All subjects gave their informed consent for inclusion before they participated in the study. The study was conducted in accordance with the Declaration of Helsinki, and the protocol was approved by the Ethics Committee of Sapienza University of Rome (prot. 1030/17).

## 4. Conclusions

**Scheme 1:**
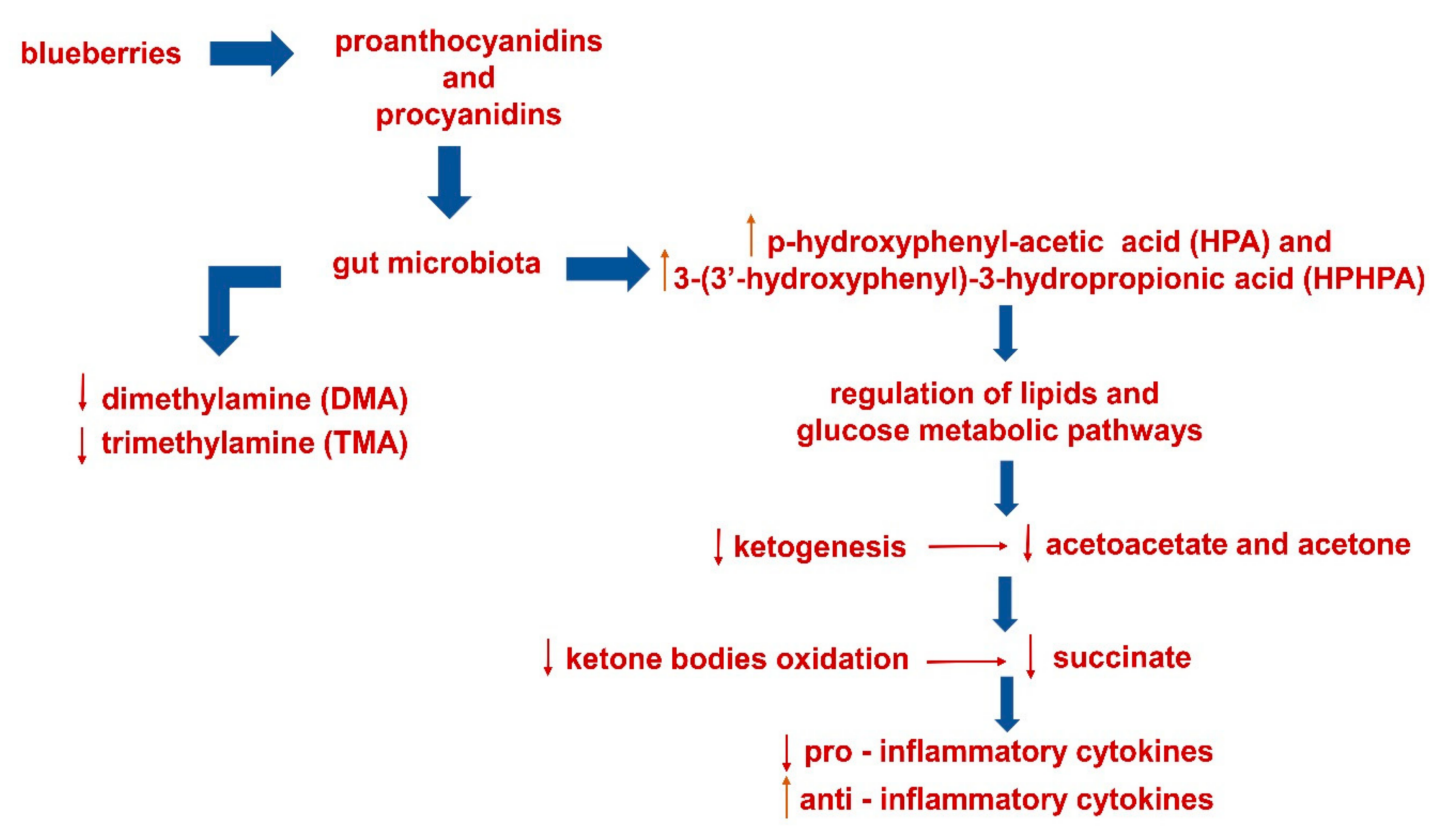
Metabolic impact of blueberries (included in a single high fat/high glycemic meal) on urinary and blood postprandial samples.

This pilot study suggests potential markers of the metabolic impact of high fat/high glycemic load meals including blueberries on patients suffering from metabolic syndrome (Scheme 1). 

Note that acetoacetate and acetone, succinate, TMA and DMA, known markers of type 2 diabetes mellitus [9], decrease after meals with blueberries. 

Moreover, acute supplementation with blueberries seems to impact the postprandial inflammation response to the experimental meal in abdominally obese adults with metabolic syndrome. In fact, the blood analysis concerning the mRNAs expression of cytokines has shown significant decrease of pro-inflammatory IL-6 and increase of anti-inflammatory TGF-β cytokines after blueberries supplementation. 

Therefore, these preliminary results suggest the combined analysis of urine metabolomes and clinical markers as a promising approach in the monitoring of the metabolic impact of blueberries. These first results would need to be further verified by wide clinical studies.

## Figures and Tables

**Table 1:** Description of recruited subjects.

Subject.	Subject 1	Subject 2	Subject 3	Subject 4	Subject 5
**Gender**	F	M	F	F	F
**Age (y)**	52	52	61	25	31
**Height (cm)**	156	174.5	158	170	152
**Weight (kg)**	87.1	90.1	71.7	84.9	92.8
**BMI (kg/m^2^)**	35.78	29.58	28.71	29.37	40.17
**WC (cm)**	103.0	100.5	92.5	91.0	115.0
**Z_50kHz_ (** **Ω** **)**	497	431	589	564.8	509.8
**PhA (°)**	7.0	6.7	5.1	6.2	6.7
**Rz_50kHz_ (** **Ω** **)**	493.3	428.1	586.7	561.5	506.3
**Xc_50kHz_ (** **Ω** **)**	60.6	43.7	52.4	61.4	59.6
**FM (kg)**	36.9	21.6	27.6	33.6	44.2
**FM %**	42.4	24.0	38.5	39.5	47.6
**FFM (kg)**	50.2	68.5	44.1	51.3	48.6
**FFM %**	57.6	76	61.5	60.5	52.4
**FFMI (kg/m^2^)**	20.6	22.5	17.7	17.8	21
**FMI (kg/m^2^)**	15.2	7.1	11.1	11.6	19.1
**FM (kg)/FFM (kg)**	0.74	0.32	0.63	0.65	0.91
**TBW (L)**	36.6	50.3	32.4	37.6	35.4
**ECW (L)**	15.2	21.5	16.3	16.8	15.1
**ICW (L)**	21.6	28.8	16.1	20.8	20.3
**BCM (kg)**	29.3	39.1	21.6	28.2	27.8
**SM (kg)**	23.3	33.8	17.8	23.9	21.2
**SMI (kg/m^2^)**	9.6	11.1	7.1	8.3	9.2
**ASMM (kg)**	20.5	27.5	15.9	20.8	19.9
**ASMM/height^2^ (kg/m^2^)**	8.42	9.03	6.37	7.20	8.61
**ASMM/weight**	0.2354	0.3052	0.2218	0.2450	0.2144

BMI = Body Mass Index; WC = Waist Circumference; Z = impedance; PhA = Phase Angle; Rz = resistance; Xc = reactance; FM = Fat Mass; FFM = Fat Free Mass; TBW = Total Body Water; ECW = Extra-Cellular Water; ICW = Intra-Cellular Water; FFMI = Fat Free Mass Index; FMI = Fat Mas Index; SM= Skeletal Muscle mass (Janssen); SMI = Skeletal Muscle mass Index; ASMM = Appendicular Skeletal Muscle Mass.

**Table 2:** Compositions of the A (with blueberries) and B (without blueberries) Meals.

**“A” Meal**		**Protein (g)**	**Carbohydrates (g)**	**Lipids (g)**	**Calories (Kcal)**
Potatoes	400 g	8.4	71.6	4.0	338.1
Bread	50 g	4.5	28.8	0.95	134.6
Baked Ham	60 g	9.4	1.0	4.6	82.6
“Mozzarella” Cheese	60 g	11.2	0.4	11.7	151.8
Butter	20 g	0.16	0.22	16.7	151.6
“Parmigiano” Cheese	10 g	3.35	0	2.8	38.7
Blueberries	150 g	1.35	7.65	0.3	36.8
Total		38.4(16.4%)	109.7(44%)	41(39.5%)	934.01
			*CHOs* (13.6 g)	*SFA* (21.6 g)	GL (70)
			*CHO* (86.4 g)	*MUFA* (3.8 g)	
				*PUFA* (11.2 g)	
**“B” Meal**		**Protein (g)**	**Carbohydrates (g)**	**Lipids (g)**	**Calories (Kcal)**
Potatoes	400 g	8.4	71.6	4.0	338.1
Bread	60 g	5.4	34.6	1.14	161.5
Baked Ham	60 g	9.4	1.0	4.6	82.6
“Mozzarella” Cheese	60 g	11.2	0.4	11.7	151.8
Butter	20 g	0.16	0.22	16.7	151.6
“Parmigiano” Cheese	10 g	3.35	0	2.8	38.7
Total		37.95(16.4%)	107.8(43.8%)	41(40.89%)	924.14
			*CHOs* (6.7 g)	*SFA* (21.6 g)	GL (70)
			*CHO* (90.9 g)	*MUFA* (3.6 g)	
				*PUFA* (11.2 g)	

CHO = Total Carbohydrate; CHOs = Sugar; GL = Glycemic Load; MUFA = Monounsaturated Fatty Acid; PUFA = Polyunsaturated Fatty Acid; SFA = Saturated Fatty Acid.

**Table 3:** ANOVA results on 75 variables to differentiate urinary samples after meals with and without blueberries. Significant variables (*p*-level < 0.05) together with the corresponding assignment are reported.

Variable/(Spectral Range)	Assignment	F	*p*-Level
V60 (2.17–2.21) ppm	Acetoacetate/Acetone	16.5	0.00091
V57 (2.39–2.40) ppm	Succinate	6.3	0.02332
V49 (2.72–2.74) ppm	Dimethylamine (DMA)	6.0	0.02614
V47 (2.91–2.94) ppm	Trimethylamine (TMA)	6.2	0.02383
V25 (6.85–6.89) ppm	3-(3’-Hydroxyphenyl)-3-hydropropionic acid/ p-Hydroxyphenyl-acetic acid	4.7	0.04517
V21 (7.14–7.22) ppm	3-(3’-Hydroxyphenyl)-3-hydropropionic acid/ p-Hydroxyphenyl-acetic acid/histidine	7.5	0.01442

**Table 4:** Two-way ANOVA uncorrected Fisher’s Least Significant Difference (LSD) test in mRNAs for pro-inflammatory cytokines (IL-1β, IL-6, TNF -α), and for anti-inflammatory cytokines (IL-4, IL-10 and TGF-β).

Cytokines	*p*-Level
IL-1β	0.807
IL-6	0.0014
TNF-α	0.5824
IL-10	0.9807
IL-4	0.0809
TGF-β	0.0038

**Table 5:** The primers for PCR amplification.

Gene	Forward Primer (5′–3′)	Reverse primer (5′–3′)
hIL1 β	GCTTATTACAGTGGCAATGAGG	GGTGGTCGGAGATTCGTAG
hIL6	GGTACATCCTCGACGGCATCT	GTGCCTCTTTGCTGCTTTCAC
hTNFα	ATCTTCTCGAACCCCGAGTGA	CGGTTCAGCCACTGGAGCT
hIL4	ACTGCACAGCAGTTCCACAG	CTCTGGTTGGCTTCCTTCAC
hIL10	GATGCCTTCAGCAGAGTGAA	GCAACCCAGGTAACCCTTAAA
hTGF β	GCAGAGCTGCGTCTGCTGAGGC	CCCGTTGATGTCCACTTGCAGTG
hGAPDH	ACAGTCAGCCGCATCTTC	GCCCAATACGACCAAATCC

## Supplementary Materials

The following are available online at https://www.mdpi.com/2218-1989/9/7/138/s1, Table S1: A total of 75 chemical shift bins and relative range (ppm) in which ^1^H-NMR spectrum has been divided.
