# Supplementary material for: Blueberry-Based Meals for Obese Patients with Metabolic Syndrome: A Multidisciplinary Metabolomic Pilot Study
Source: Metabolites. 2019 Jul 10;9(7):138. doi: 10.3390/metabo9070138 (PMC6680695; doi:10.3390/metabo9070138)
Supplement: Supplementary file 1 [file metabolites-09-00138-s001.pdf]

Supplementary Material

For

Blueberries–meals for obese patients with metabolic syndrome: a multidisciplinary metabolomic pilot study

Anatoly Petrovich Sobolev [1], Alessandra Ciampa [2], Cinzia Ingallina [2*], Luisa Mannina [2,1*], Donatella Capitani [1], Ilaria Ernesti [3], Elisa Maggi [4], Rita Businaro [4], Maria Del Ben [5], Petra Engel [6], Anna Maria Giusti [3], Lorenzo M. Donini [3], Alessandro Pinto [3]

1 Istituto per i Sistemi Biologici, CNR, Laboratorio di Risonanza Magnetica "Annalaura Segre", via Salaria km 29.300, I–00015 Monterotondo, Italia; anatoly.sobolev@cnr.it (A.P.S.); donatella.capitani@cnr.it (D.C)

2 Dipartimento di Chimica e Tecnologie del Farmaco, Sapienza Università di Roma, Piazzale Aldo Moro 5, I–00185 Roma, Italia; luisa.mammina@uniroma1.it (L.M.); cinzia.ingallina@uniroma1.it (C.I.); alessandra.ciampa@uniroma1.it (A.C.)

3 Sezione di Fisiopatologia Medica, Scienza dell'Alimentazione ed Endocrinologia – Dipartimento di Medicina Sperimentale, Sapienza Università di Roma, Piazzale Aldo Moro 5, I–00185 Roma, Italia; alessandro.pinto@uniroma1.it (A.P.); ilariaernesti@libero.it (I.E.); annamaria.giusti@uniroma1.it (A.M.G.); lorenzomaria.donini@uniroma1.it (L.D.)

4 Dipartimento di Scienze e Biotecnologie medico–chirurgiche, Sapienza Università di Roma, Corso della Repubblica 79, 04100 Latina, Italia; rita.businaro@uniroma1.it (R.B.); elisa.maggi@uniroma1.it (E.M.)

5 Dipartimento di Medicina Interna e Specialità Mediche, Policlinico Umberto 1 Sapienza Università di Roma, viale del Policlinico 151, I–00185, Roma, Italia; maria.delben@uniroma1.it (M.D.B.)

6 Consiglio per la Ricerca in Agricoltura e l'Analisi dell'Economia Agraria, Ufficio Rapporti Istituzionali e Relazioni Internazionali, via Po 14, 00198 Roma, Italia; petra.engel@crea.gov.it (P.E.)

* Correspondence: luisa.mannina@uniroma1.it (L.M.); cinzia.ingallina@uniroma1.it (C.I.)
Tel. +39–06–4991–3735

Table of content

Supplementary Table S1. Seventy five chemical shift bins and relative range (ppm) in which ^{1}H–NMR spectrum has been divided.

Bin	Range (ppm)
V2	9.390–9.329
V3	9.299–9.251
V4	9.144–9.092
V5	9.012–8.933
V6	8.868–8.812
V7	8.806–8.765
V8	8.690–8.627
V9	8.550–8.480
V10	8.470–8.416
V11	8.408–8.010
V12	8.008–7.780
V13	7.688–7.671

V14	7.670–7.616
V15	7.586–7.526
V16	7.453–7.402
V17	7.388–7.346
V18	7.336–7.308
V19	7.308–7.286
V20	7.285 –7.257
V21	7.224–7.139
V22	7.138–6.953
V23	6.940–6.905
V24	6.905–6.888
V25	6.886–6.852
V26	6.852–6.836
V27	6.684–6.669
V28	6.669–6.636
V29	6.621–6.592
V30	5.521–5.285
V31	5.285–5.229
V32	5.229–4.894
V34	4.472–4.423
V35	4.093–4.042
V36	3.989–3.954
V37	3.583–3.565
V38	3.458–3.447
V39	3.447–3.421
V40	3.376–3.357
V41	3.301–3.261
V42	3.238–3.221
V43	3.214–3.187
V44	3.174–3.137
V45	3.130–3.106
V46	3.075–3.024
V47	2.945–2.914
V48	2.884–2.865
V49	2.743–2.718
V50	2.718–2.697
V51	2.695–2.671
V52	2.571–2.545
V53	2.544–2.518
V54	2.492–2.428
V55	2.428–2.415
V56	2.415–2.401
V57	2.401–2.386
V58	2.365–2.318
V59	2.297–2.246
V60	2.210–2.167
V61	2.115–2.014

V62	2.014–1.968
V63	1.692–1.609
V64	1.507–1.472
V65	1.447–1.428
V66	1.376–1.355
V67	1.356–1.319
V68	1.285–1.266
V69	1.267–1.255
V70	1.254–1.223
V71	1.214–1.177
V72	1.176–1.146
V73	1.146–1.131
V74	1.126–1.093
V75	1.092–1.059
V76	1.058–1.034
V77	1.014–0.812
